# The Impact of In‐Hospital Disease Modifying Treatments on Mental and Physical Burden in Caregiver of Patients With MS

**DOI:** 10.1002/acn3.70026

**Published:** 2025-03-10

**Authors:** Giuseppe Schirò, Salvatore Iacono, Michele Andolina, Gabriele Sorbello, Andrea Calì, Erika Gentile, Marco D'Amelio, Paolo Aridon, Giuseppe Salemi, Paolo Ragonese

**Affiliations:** ^1^ Department of Biomedicine, Neurosciences and Advanced Diagnostics (BiND) University of Palermo Palermo Italy; ^2^ Neurology and Multiple Sclerosis Center, Neurology Unit Foundation Institute ‘G. Giglio’ Cefalù Italy

**Keywords:** caregiver burden of care, home‐based disease modifying treatments, mental health, multiple sclerosis, unmet need

## Abstract

**Objective:**

People with multiple sclerosis (pwMS) may require a high level of daily assistance both for indoor and outdoor activities. Usually, relatives or friends provide daily support to MS patients who have lost personal autonomy. Several factors such as disability level and disease duration may affect the burden of care in caregivers of pwMS; however, the relationship between disease‐modifying therapies (DMTs) and caregiver burden has never been explored so far. The aim of this study is to explore the impact of hospital‐based therapies on anxiety, depression, and burden of care in caregivers of pwMS.

**Methods:**

Hospital Anxiety and the Depression Scale (HADS) and Caregiver Burden Inventory (CBI) questionnaires were administered to caregivers of pwMS who performed planned visits in the outpatient setting. Multivariable regression models were built to evaluate the association between hospital‐based therapies and depression (HADS‐D > 7), anxiety (HADS‐A > 7) and need to rest (CBI > 24).

**Results:**

Caregivers of pwMS receiving in‐hospital therapies achieved higher scores in HADS and CBI questionnaires, resulting in a higher proportion of anxiety, depression, and need to rest among these. The multivariable models also showed that hospital‐based therapies were positively associated with caregivers' depression (aOR = 2.38 [1.04–5.5; *p* = 0.04]), anxiety (aOR = 2.36 [1.03–5.4; *p* = 0.043]) and need to rest (aOR = 2.06 [0.8–5.29]; *p* = 0.13).

**Interpretation:**

Hospital‐based therapies in pwMS negatively affect the burden of care and mental health of their own caregivers. The availability of home‐based highly effective DMTs may contribute to reducing the outdoor caregiver burden without renouncing highly effective treatments.

## 1 Introduction

Multiple sclerosis (MS) is an autoimmune demyelinating disease of the central nervous system (CNS) representing the leading cause of neurological disability in young people [1]. It has been shown that early treatment with highly effective DMTs (HE‐DMTs) was able to reduce disability accrual and the loss of activities of daily living (ADL) of people with MS (pwMS) [2]. However, it is not uncommon for these patients, especially those with progressive MS, to experience physical (e.g., spasticity, balance disorders, fatigue, etc) and psychiatric symptoms (e.g., anxiety, depression, etc) affecting negatively their quality of life and ADL [3, 4]. Particularly, MS progression is often characterized by inexorable loss of personal autonomy in ADL; therefore, these patients may require a high level of daily assistance for both indoors (e.g., dressing, feeding, personal hygiene, etc) and outdoor activities (e.g., walks, assignments, shopping) [5]. Commonly, pwMS who have lost autonomy in ADL are supported in their activities by relatives and/or friends who usually provide physical, emotional, and social support to them [6]. Furthermore, it has been shown that the time lost at work by caregivers spent accompanying pwMS to hospital appointments was an indirect indicator of caregiver strain [7]. Interestingly, several MS or caregiver‐related factors may affect the caregiver experience, including socio‐economic context, educational level of caregiver, and disability burden of pwMS. Accordingly, caregivers of pwMS also have a reduced quality of life as well as they commonly experience anxiety and depression [6, 8]. While the necessities of pwMS were largely explored, often the unmet needs of their caregivers are overlooked. Indeed, the efforts of caregivers of pwMS are heterogeneous; although the impact of disease‐modifying treatments (DMTs) on caregivers' burden of care and behavior has never been investigated to date. In this perspective, it is reasonable to hypothesize that hospital‐based DMTs may affect caregiver's responsibility, mental health, and quality of life subsequently. Based on these considerations, the aim of this study was to evaluate the impact of hospital‐administered DMTs compared to home‐based DMTs on burden of care, anxiety, and depression in caregivers of pwMS.

## 2 Materials and Methods

### 2.1 Study Design and Participants

This prospective cohort study was carried out by enrolling pwMS and their paired caregivers who underwent scheduled neurological visits to our MS Clinic, University Hospital, Palermo, Italy, between 01st December 2022 and 30th September 2023. The inclusion criteria for both pwMS and caregivers were willingness to participate and signed written informed consent while only caregivers who met the definition of primary caregivers (i.e., those who cared for the patient most of the time) were enrolled. Exclusion criteria were lack of informed consent form, inability to fulfill the questionnaire, incomplete questionnaires failing to meet the definition of primary caregiver, and neurological examination beyond the enrollment period.

### 2.2 Data Collection

Demographic and clinical features of pwMS, including disease duration, disability burden, and clinical course, were routinely collected during the outpatient visit. The grade of disability was evaluated through the Expanded Disability Status Scale (EDSS) [9]. Meanwhile, while the MS patient was undergoing a neurologic examination, in a separate room, their paired caregiver was asked to fill in with a pen the printed version of the Hospital Anxiety and Depression Scale (HADS) and Caregiver Burden Inventory (CBI) questionnaires [10, 11]. In addition, we provided each caregiver with a printed form that included the collection of their demographic information (i.e., sex, age, education) and whether pwMS and their paired caregiver were cohabitants.

### 2.3 Study Assessments

HADS and CBI questionnaires were administered and self‐fulfilled by caregivers of pwMS. Subsequently, we reviewed the questionnaires and calculated the HADS sum score, HADS‐depression (HADS‐D) and HADS‐anxiety (HADS‐A) subscores; CBI sum score and its subitem scores, including time‐dependence, developmental, physical, emotional, and social items, were also calculated. An achieved score ≥ 8 at HADS‐D and HADS‐A was used to define the presence of depression and anxiety, respectively, according to literature [10]. The cut‐off values of 24 and 36 obtained at CBI were used to identify the need to rest (NTR) and risk of burning out (RBO), respectively [11].

### 2.4 Defining Home‐Based and In‐Hospital Therapies

Participants included in this study were divided into two groups: those receiving home therapy and others receiving in‐hospital therapies. The in‐hospital therapies included those DMTs that were inevitably administered in a hospital setting, including natalizumab, ocrelizumab, alemtuzumab, and rituximab. Conversely, the home‐based therapies included oral or self‐administered injectable DMTs such as ofatumumab, cladribine, sphingosine 1‐phosphate receptor modulators, dimethyl fumarate, teriflunomide, glatiramer acetate, or interferon compounds. For the analyses purpose, patients who did not take any DMTs at the time of data collection were considered as receiving home‐based therapy since they usually received symptomatic MS drugs.

### 2.5 Statistical Analysis

Shapiro–Wilks test was used to check for the normal distribution of quantitative variables. These were reported as median and interquartile ranges (IQR) or as mean and standard deviation (SD) and analyzed by using Student's *T* test or Mann and Whitney test, as appropriate. Qualitative variables were reported as number and relative percentage and analyzed by using Chi squared test or Fisher's exact test, accordingly. To evaluate the predictors of anxiety, depression, and NTR, an unconditional logistic regression analysis was performed for each study variable by considering the presence of each variable as an independent variable. The odds ratios (OR) with 95% confidence intervals (CI) and two‐tailed *p* value (alpha < 0.05) were calculated. Multivariable regression analysis was performed to obtain the independent effect of hospital‐based therapies on study endpoints by adjusting for one or several other factors. All the predictors with a statistical significance level lesser than or equal to 0.1 were included in the multivariable model while age and sex of caregivers were considered as a priori confounders. Statistical analyses were performed by using the Statistical Package for the Social Sciences software (IBM SPSS Statistics, Version 26.0; 2019. Armonk, NY: IBM Corp).

### 2.6 Ethics Statement

All the participants signed the written informed consent form to participate in this study. The study was conducted in accordance with the Declaration of Helsinki and was approved by the local Institutional Review Board (Protocol code number 10/2019).

## 3 Results

**FIGURE 1:**
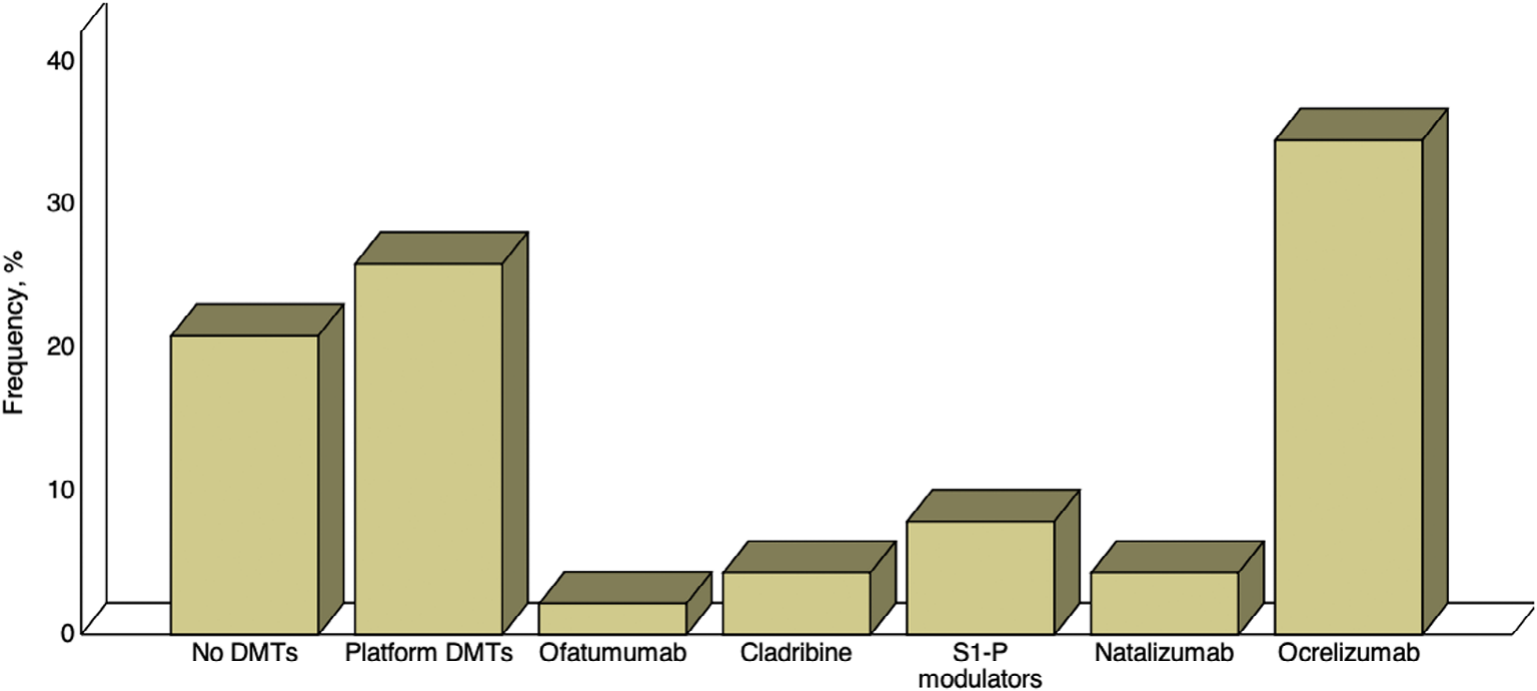
Bar‐charts reporting the relative frequency of home and in‐hospital therapies among patients with multiple sclerosis included in this study. DMTs, disease‐modifying treatments; S1‐P, sphingosine 1‐phosphate.

All the continuous variables had a non‐normal distribution (all *p* < 0.05). Out of 139 pwMS enrolled in this study, *n* = 86 (61.9%) were receiving home therapies, while *n* = 54 (38.8%) were receiving in‐hospital therapy. Platform DMTs (*n* = 36; 42.4%) were the most represented home therapies, while ocrelizumab was the most used in‐hospital DMTs (Figure 1).

The median MS duration was 8 years, the proportion of patients with progressive MS course reached 44.6% (*n* = 62) while the median age of caregiver was 53 years; we did not find statistically significant differences when comparing demographic and clinical factors of pwMS and their caregivers among groups at the exception of the proportion of pwMS with an EDSS equal to or higher than four in the hospital‐therapy group (Table 1).

**TABLE 1:** Demographic and clinical features of patients with Multiple Sclerosis and their paired caregivers included in this study.

	Home therapy *N* = 85	In hospital therapy *N* = 54	Total *N* = 139	*p*
MS Patients				
Women, *n* (%)	61 (71.8)	43 (79.6)	104 (74.8)	0.30
Age at the evaluation day y, median (IQR)	49 (38–60)	45 (33–56)	48 (35–58)	0.11
MS duration, median (IQR)	7.9 (3–18)	7.9 (4–13)	7.9 (3.5–17)	0.84
MS duration > 7 years, *n* (%)	47 (55.3)	30 (55.6)	77 (55.4)	0.98
EDSS, median (IQR)	3 (1.5–6.5)	6 (3.5–6.5)	5 (1.5–6.5)	0.11
EDSS ≥ 4	42 (49.4)	40 (74.1)	82 (59)	0.004
Progressive MS course, *n* (%)	35 (41.2)	27 (50)	62 (44.6)	0.31
Caregivers				
Women, *n* (%)	43 (50.6)	20 (37)	63 (45.3)	0.12
Age y, median (IQR)	52 (42–64)	53.5 (44–60)	53 (43–63)	0.94
Education, median (IQR)	13 (8–13)	13 (8–13)	13 (8–13)	0.13
Cohabitant, *n* (%)	70 (82.4)	50 (92.6)	120 (86.3)	0.09

Abbreviations: EDSS, Expanded Disability Status Scale; IQR, interquartile range; MS, multiple sclerosis.

**FIGURE 2:**
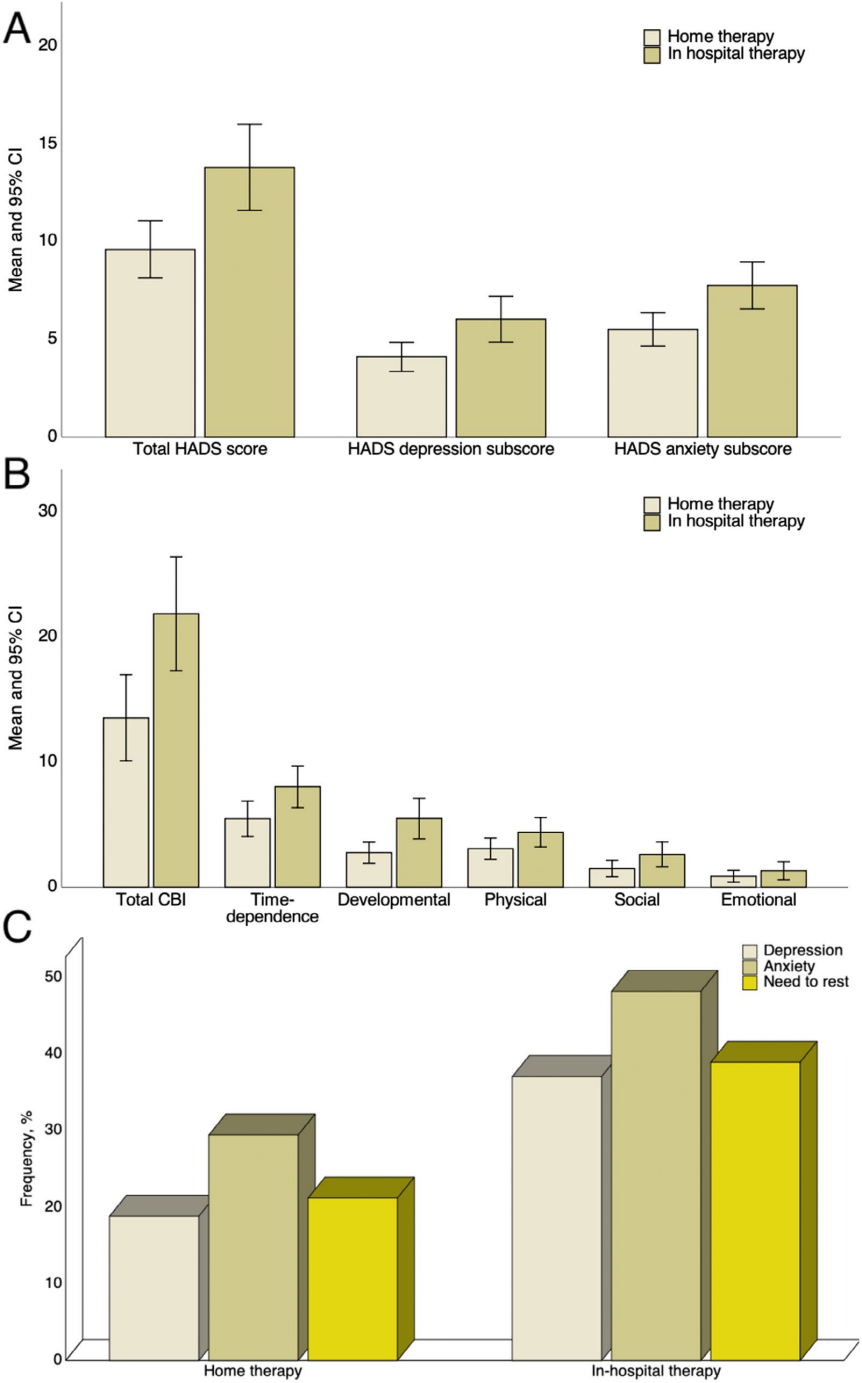
Bar‐charts showing the comparison of total HADS, HADS‐D, and HADS scores (A), CBI and its subitems scores (B), and relative frequency of anxiety, depression, and need to rest (C) between caregivers of pwMS receiving home‐ and in‐hospital therapies. HADS, Hospital Anxiety and Depression Scale; CBI, Caregiver Burden Inventory; CI, confidence interval; pwMS, people with Multiple Sclerosis.

Caregivers of pwMS receiving in‐hospital therapies had higher total HADS scores (*p* = 0.003), HADS‐D (*p* = 0.009) and HADS‐A subscores (*p* = 0.004) compared to those of patients in the home‐therapy group, as shown in Figure 2A.

At the same manner, these patients achieved a higher total CBI score (*p* < 0.001), time‐dependency (*p* = 0.002), developmental (*p* = 0.008), physical health (*p* = 0.048) and emotional health (*p* = 0.002) subitems as depicted in Figure 2B. By categorizing data according to reference values of HADS and CBI questionnaires, we found that the caregiver of pwMS undergoing in‐hospital treatment had a higher frequency of anxiety (48.1% vs. 29.4%; *p* = 0.03), depression (37% vs. 18.6%; *p* = 0.02) and need to rest (38.9% vs. 21.2%; *p* = 0.02) as shown in Figure 2C. Full comparisons of HADS and CBI mean scores obtained from caregivers included in this study are reported in Table 2.

**TABLE 2:** Comparison of HADS and CBI scores between caregivers of pwMS receiving home and in‐hospital therapies.

	Total mean (SD)	Home therapy mean (SD)	Hospital therapy mean (SD)	Mean difference	95% CI		*p* *
					Lower	Upper	
HADS total score	11.2 (7.5)	9.5 (6.7)	13.7 (8.1)	4.2	1.7	7	0.003
Depression subscore	4.8 (3.8)	4.1 (3.4)	6 (4.3)	1.9	0.6	3.2	0.009
Anxiety subscore	6.3 (4.2)	5.5 (3.9)	7.7 (4.4)	2.2	0.8	3.7	0.004
CBI total score	16.7 (16.6)	13.5 (16)	21.8 (16.7)	8.3	2.7	13.9	< 0.001
Time dependency	6.4 (6.4)	5.5 (6.5)	8 (6.1)	2.6	0.4	4.8	0.002
Development	3.8 (4.9)	2.7 (3.8)	5.4 (5.9)	2.7	1.0	4.3	0.008
Physical health	3.5 (4.1)	3.1 (3.9)	4.3 (4.2)	1.3	−0.1	2.7	0.048
Emotional health	1.9 (3.3)	1.4 (3)	2.6 (3.6)	1.1	−0.002	2.6	0.002
Social relationship	1.04 (2.3)	0.7 (2.2)	1.3 (2.6)	0.4	−0.4	1.3	0.12

Abbreviations: CBI, caregiver burden inventory; CI, confidence interval; HADS, hospital anxiety and depression scale; MS, multiple sclerosis; SD, standard deviation.

* Mann and Whitney test.

### 3.1 Factors Associated With Caregiver's Depression

Higher EDSS and disease duration as well as progressive MS course and in‐hospital therapy were positively associated with the presence of depression in caregivers at univariate analysis; however, in‐hospital therapy singled out as statistically associated with depression (aOR = 2.38 [95% CI 1.04–5.5]; *p* = 0.04) at multivariate analysis while the education level of caregivers was inversely associated with it (aOR = 0.89 [95% CI 0.8–1.0]; *p* = 0.057) although this was not statistically significant (Table 3).

**TABLE 3:** Univariate and multivariate logistic regression model exploring predictors of caregivers' depression.

	Univariate analyses			aOR	Multivariate analyses	
	OR	95% CI	*p*		95% CI	*p*
MS patients						
Age	1.01	0.99–1.04	0.36			
Man	0.65	0.25–1.64	0.36			
EDSS ≥ 4	1.83	0.8–4.2	0.1	1.25	0.5–3.0	0.62
Disease duration > 7 years	1.37	0.6–3.0	0.42			
Progressive MS course	1.15	0.54–2.47	0.71			
Caregiver						
Age	1.01	0.98–1.04	0.54	1.01	0.98–1.04	0.74
Man	0.78	0.36–1.66	0.51	0.74	0.33–1.7	0.46
Education	0.87	0.78–0.98	0.016	0.89	0.8–1.0	0.057
Cohabitant	1.36	0.42–4.42	0.61			
In‐hospital therapies	2.54	1.2–5.5	0.019	2.38	1.04–5.5	0.04

Abbreviations: EDSS, Expanded Disability Status Scale; MS, Multiple Sclerosis.

### 3.2 Factors Associated With Caregiver's Anxiety

The univariate logistic regression showed that a higher age of patients, a higher EDSS, a longer disease duration, and a progressive MS course were associated with the presence of anxiety in caregivers (Table 4). At the multivariate analyses, in‐hospital therapies remained the only factor positively and statistically significantly associated with anxiety in caregivers (aOR = 2.36 [95% CI 1.03–5.4]; *p* = 0.043) while no other relevant associations were found (Table 4).

**TABLE 4:** Univariate and multivariate logistic regression model exploring predictors of caregivers' anxiety.

Predictors	Univariate analyses			aOR	Multivariate analyses	
	OR	95% CI	*p*		95% CI	*p*
MS patients						
Age	1.02	0.99–1.05	0.09	1.02	0.98–1.06	0.27
Man	1.42	0.65–3.1	0.38			
EDSS ≥ 4	2.92	1.37–6.22	0.005	1.64	0.62–4.32	0.32
Disease duration > 7 years	1.83	0.9–3.7	0.09	1.39	0.6–3.24	0.45
Progressive MS course	1.70	0.85–3.42	0.13			
Caregiver						
Age	0.99	0.97–1.02	0.83	0.99	0.95–1.01	0.30
Man	0.62	0.31–1.23	0.17	0.74	0.33–1.7	0.46
Education	0.91	0.82–0.99	0.04	0.93	0.84–1.04	0.19
Cohabitant	0.99	0.36–2.71	0.98			
In‐hospital therapies	2.23	1.1–4.53	0.027	2.36	1.03–5.40	0.043

Abbreviations: EDSS, Expanded Disability Status Scale; MS, Multiple Sclerosis.

### 3.3 Factors Associated With Need to Rest

Although older age, higher EDSS, progressive MS course, longer MS duration, and being a cohabitant of pwMS were significantly associated with the need to rest at univariate analysis, none of these factors reached the statistical significance level after performing multivariate analyses, although being a cohabitant reached the highest strength of association (aOR = 3.49 [95% CI 0.7–18]; *p* = 0.14) as reported in Table 5.

**TABLE 5:** Univariate and multivariate logistic regression model exploring predictors of caregivers' need to rest.

	Univariate analyses			aOR	Multivariate analyses	
	OR	95% CI	*p*		95% CI	*p*
MS patients						
Age	1.05	1.02–1.08	0.003	1.03	0.99–1.07	0.17
Female	0.85	0.36–2.04	0.72			
EDSS ≥ 4	7.36	2.66–20.4	< 0.0001	2.60	0.65–10.5	0.18
Disease duration > 7 years	2.25	1.03–9.93	0.043	1.18	0.45–3.11	0.73
Progressive MS course (relapsing course ref.)	4.94	2.19–11.1	< 0.0001	1.93	0.65–5.77	0.24
Caregiver						
Age	1.01	0.98–1.04	0.40	0.99	0.95–1.02	0.56
Male	0.96	0.45–2.01	0.90	0.94	0.40–2.21	0.88
Education	0.89	0.81–0.99	0.036	0.94	0.83–1.07	0.34
Cohabitant	3.79	0.83–17.3	0.085	3.49	0.67–17.95	0.14
In‐hospital therapies	2.37	1.11–5.04	0.025	2.06	0.80–5.29	0.13

Abbreviations: DMT, disease modifying treatments; EDSS, Expanded Disability Status Scale; MS, Multiple Sclerosis.

## 4 Discussion

In this prospective cohort study, we evaluated the impact of hospital‐based DMTs on caregivers' burden, anxiety and depression by evaluating HADS and CBI questionnaires. Most of the existing studies investigated the clinical and demographic features of caregivers of pwMS although the evaluation of the determinants of caregivers strain and mental health is still poorly explored [12]. Firstly, we found that caregivers of pwMS receiving in‐hospital DMTs achieved higher scores at HADS (Figure 2A) and CBI (Figure 2B) questionnaires compared to those receiving home‐therapy (Table 2). This turned out in a higher proportion of caregiver with anxiety (48.1%), depression (37%) and NTR (38.9%) in the in‐hospital group when compared to caregivers of pwMS receiving home‐based therapy. When analyzing factors associated to anxiety, depression and need to rest in caregiver of pwMS in our cohort, we found that caregiver of pwMS receiving in‐hospital therapy had a higher probability to develop depression (Table 3), anxiety (Table 4) and NTR (Table 5) compared to caregivers of pwMS receiving hospital‐therapy. Although the moderate association between in‐hospital therapy and NTR, this result did not turn our statistically significant but this may be due to the lower number of enrolled participants in our study. Interestingly, being cohabitant of pwMS increased the odds of NTR (Table 5) while it did not affect neither anxiety nor depression. No other MS‐related factors, such as progressive MS course and disease duration were significantly associated to depression and anxiety while each year increase of caregiver education was associated with a 13% lower probability to develop depression (Table 3). The efforts of caregivers of pwMS are several. First, they may support emotionally and/or physically pwMS during both indoor and outdoor activities [13]. Secondly, according to MS progression and the gradual loss of autonomy, the caregivers may be also engaged in daily personal care activities such as feeding, dressing and personal hygiene [13, 14]. Third, they accompany pwMS to the clinic where they often encounter long and frustrating waits. Moreover, caregivers of pwMS who are unable to drive or living in rural areas are also burdened by an exaggerated sense of responsibility since delayed treatment may result in MS worsening [7]. In this view, caregivers of pwMS may also have an indirect role on adherence of treatment and clinical outcomes consequently. Our results highline how in‐hospital therapy in pwMS may contribute to increase the caregiver's time‐dependence, developmental, physical, and social burden. However, our findings need clarification. The majority of patients in the hospital‐based therapies group were receiving ocrelizumab, turning out in a putative frequency of two hospital admissions per year. However, these patients periodically underwent several medical assessments and laboratory testing preparatory to infusion, which are usually carried out at our clinic in a hospital setting, resulting in a higher‐than‐expected hospital admission in these patients. In addition, patients receiving natalizumab usually come to the clinic 10–12 times per year. Finally, in‐hospital therapy usually requires long administration time, which may also negatively affect caregiver's behavior. Based on these premises, it is simply to hypothesize that the number of planned infusions, the responsibility of treatment adherence, the need to perform preparatory medical assessments and the lengthy waiting time at the hospital may be the factor affecting time‐dependance, physical strain, social activities and mental behavior in caregiver of pwMS. Thus, being caregiver of pwMS may be exhausting, particularly of pwMS receiving in‐hospital therapies. In this perspective, targeted interventions aimed to reducing caregiver strain and burden of care may be driven by home therapies. In this sense, home therapies, both platform and HE‐DMTs, may have less impact on burden of care in caregiver of pwMS since their management is entrusted entirely to the patient. It is worth nothing that home therapy does not mean less effectiveness. Indeed, the increasing availability of home‐administered HE‐DMTs, both oral (e.g., cladribine, sphingosine 1‐posphate modulators, etc) and injective (e.g., ofatumumab, etc), may contribute to reducing the outdoors caregiver burden without depriving pwMS from higly effective therapy. On the other hand, the reduction of the time spent inside the hospital as well as the reduction of the number of hospital admissions by overlapping treatment administration, laboratory testing and clinical examination in a single appointment, may also contribute to reducing caregiver strain. Recently, indeed, results from the EASIER study underlined the importance of the shorter hospitalization time in pwMS treated with subcutaneous natalizumab [15]. Our study has some limitations. Firstly, we enrolled a lower number of participants, and this may have prevented the achievement of statistical significance of such predictors at multivariable regression models. At the same time, although the statistical significance was not reached, the magnitude of the association between predictors and outcome variable should not be overlooked since many OR indicated a moderate to higher association which could be clinically relevant. Secondly, although we conducted a prospective study, the selection bias cannot be ruled out since many patients were not accompanied by their caregiver even though they have one; therefore, these patients and their caregivers were obviously not included in this study. Finally, we did not take into account anxiety and depression scores of pwMS because this was beyond the objectives of our study although the evalutation of patient's perspective may be interesting in future studies.

## 5 Conclusion

The in‐hospital therapies of pwMS may negatively affect time‐dependency, physical health, emotional health, and mental health of their own caregivers, while other factors such as disability, progressive MS course, and disease duration seem to have less impact on caregiver burden. Highly effective home‐based DMTs, together with the reduction of the time spent in the hospital, may be successful strategies for reducing hospitalization of pwMS, with a positive reframing of the burden of care in their own caregiver and concurrently without depriving pwMS of HE‐DMTs. Our findings also stimulate the development of new strategies with the aim to reduce caregiver strain, focusing not only on unmet needs of pwMS but also on their family network.

## Author Contributions

Conceptualization, study design, and methodology: Giuseppe Schirò, Salvatore Iacono, Giuseppe Salemi, and Paolo Ragonese. Data collection: Michele Andolina, Gabriele Sorbello, Andrea Calì, Erika Gentile, Giuseppe Schirò, and Salvatore Iacono. Data analyses: Salvatore Iacono, Giuseppe Schirò, and Paolo Ragonese. Writing – original draft preparation: Giuseppe Schirò and Salvatore Iacono. Manuscript revision and substantial editing: Paolo Ragonese, Paolo Aridon, Marco D'Amelio, and Giuseppe Salemi. All authors have read and agreed to the published version of the manuscript.

## Consent

Informed consent was obtained from all subjects involved in the study.

## Conflicts of Interest

The authors declare no conflicts of interest.

## Data Availability

Data are available upon reasonable request to the corresponding author.
